# Use of FreeStyle Libre Flash Monitor Register in the Netherlands (FLARE-NL1): Patient Experiences, Satisfaction, and Cost Analysis

**DOI:** 10.1155/2019/4649303

**Published:** 2019-11-03

**Authors:** M. J. Fokkert, A. Damman, P. R. van Dijk, M. A. Edens, S. Abbes, J. Braakman, R. J. Slingerland, L. D. Dikkeschei, J. Dille, H. J. G. Bilo

**Affiliations:** ^1^Isala, Department of Clinical Chemistry, Zwolle, Netherlands; ^2^Isala, Department of Innovation and Science, Zwolle, Netherlands; ^3^Isala, Diabetes Research Center, Zwolle, Netherlands; ^4^Isala, Department of Internal Medicine, Zwolle, Netherlands; ^5^University of Groningen, University Medical Center Groningen, Department of Internal Medicine, Groningen, Netherlands

## Abstract

In patients with diabetes mellitus (DM), adequate glucose control is of major importance. When treatment schemes become more complicated, proper self-management through intermittent self-measurement of blood glucose (SMBG), among others, becomes crucial in achieving this goal. In the last decade, continuous glucose monitoring (CGM) has been on the rise, providing not only intermittent information but also information on continuous glucose trends. The FreeStyle Libre (FSL) Flash CGM system is a CGM system mainly used for patients with DM and is designed based on the same techniques as early CGMs. Compared with earlier CGMs, the FSL is factory calibrated, has no automated readings or direct alarms, and is cheaper to use. Although less accurate compared with the gold standard for SMBG, users report high satisfaction because it is easy to use and can help users monitor glucose trends. The Flash Monitor Register in the Netherlands (FLARE-NL) study aims to assess the effects of FSL Flash CGM use in daily practice. The study has a before-after design, with each participant being his or her own control. Users will be followed for at least 1 year. The endpoints include changes in HbA1c, frequency and severity of hypoglycemias, and quality of life. In addition, the effects of its use on work absenteeism rate, diabetes-related hospital admission rate, and daily functioning (including sports performance) will be studied. Furthermore, cost-benefit analysis based on the combination of registered information within the health insurance data will be investigated. Ultimately, the data gathered in this study will help increase the knowledge and skills of the use of the Flash CGM in daily practice and assess the financial impact on the use of the Flash CGM within the Dutch healthcare system.

## 1. Introduction

In individuals with diabetes mellitus (DM), it has been unanimously accepted that the development of micro- and macrovascular complications is linked to the duration and severity of hyperglycemia [1]. However, besides hyperglycemia, the presence of fluctuations in blood glucose concentrations (so-called “glycemic variability”) is also associated with the risk of microvascular complications and hypoglycemia with subsequent stress, anxiety, impaired health, and reduced quality of life (QoL) [2, 3]. Therefore, efforts are made to maintain blood glucose levels as close to normal as possible, while minimizing glycemic variability and, consequently, the risk of hypoglycemia.

At present, blood glucose concentrations are mainly measured by applying a drop of blood to a chemically treated “test strip,” which is then inserted into an electronic blood glucose meter (BGM). In the last few decades, BGMs have been supplemented and even supplanted by systems measuring interstitial fluid, aiming at “real-time” continuous glucose measurements (rt-CGM) [4, 5] with and without intelligent connection with an external insulin pump. However, measuring and assessing interstitial fluid glucose levels and translating such outcomes into results comparable to capillary glucose concentrations have been a challenge [6, 7].

In 2014, the FreeStyle Libre Flash Monitor (FSL-FM) system [8] was introduced, which performs on partially different principles than earlier CGMs. The user must proactively obtain the results by using a reader instead of data being relayed automatically to a receiver. Furthermore, the FSL-FM is calibrated during the fabrication process instead of being calibrated daily by the patient and is meant to be inserted in the upper arm only. According to the manufacturer, no further individual calibration is needed, not to mention that the patient does not have access to the monitor to be able to do so. Use of the FSL-GM reduces, but does not remove, the need for BGM.

In the light of the challenges to acquire reliable and accurate data, our research group assessed the performance of the FSL-FM by conducting an independent validation study [9]. We demonstrated a high correlation between the gold standard isotope dilution gas chromatograph-mass spectrometer (ID-GCMS) and the capillary measurement techniques used in our study (SMBG). The FSL-FM was less accurate compared with the SMBG and also the gold standard. Under normal circumstances, glucose levels on the low end of the spectrum as shown by the FSL-FM are generally lower than the results provided by other techniques including the gold standard, while glucose levels on the high end are generally recorded higher. Furthermore, after a standardized meal, glucose levels assessed by the FSL-FM rose slower compared with the results of all other tested methods for glucose measurement.

Although the FSL-FM is less accurate in measuring blood glucose levels compared with other methods, the overall patient satisfaction with the FSL, both in individual user experiences and in studies, is high. FSL users were better informed of their glucose trends and levels and were more confident in their decisions. Hence, from a user perspective, this new device is very much appreciated because of its ease of use, the possibility to see trends in glucose changes, and the possibility to obtain 24-hour general information for the last 14 days, provided the readings were performed at least every 8 hours.

Despite the high patient satisfaction with the new system, the Dutch health authorities and insurance companies considered the scientific evidence and technical aspects of the FSL-FM to be insufficient to warrant widespread reimbursement. Furthermore, they considered the description of specific target groups as being too diffuse. Hence, the Flash Monitor Register in the Netherlands (FLARE-NL) was established to answer these concerns. On the contrary, both the Dutch diabetes patient organization (Diabetesvereniging Nederland, DVN) and some Dutch healthcare providers welcomed the FSL-FM as an adjunct to present glucose measurement possibilities. The main arguments by both groups are the individually reported improvements in QoL and reduction in the users' fear of unexpected hypoglycemia. Furthermore, the ability to see trends is considered a major positive asset by users.

This before and after interventional study aimed to assess the patient-oriented endpoints related to the use of the FreeStyle Libre Flash response system (FSL-FM) in persons with diabetes mellitus (Flash Monitor Register in the Netherlands: FLARE-NL) in daily practice.

## 2. Materials and Methods

### 2.1. Aims

The register aimed to assess the effects of the FSL-FM on user-related endpoints.

Primary outcome measures are changes in hemoglobin A1c (HbA1c), QoL using the 12-Item Short-Form Health Survey (SF-12) [10] and the six-dimensional EuroQol instrument (EQ-6D) [11–13], the number of hypoglycemic episodes, and the number of hospitalizations related to DM. Secondary outcomes include changes in the number of working days lost/days absent from work due to illness, daily functioning (including sports performance), and results of cost-effectiveness and cost-benefit analyses of combining register and health insurance data.

We hypothesize that the use of the FSL-FM will improve glycemic control (especially in those patients with the worst metabolic control) and QoL; its effects on diabetes-related hospitalizations will probably be minimal. We expect a decrease in work absenteeism rate and an increase in exercise activity, possibly related to increased self-confidence.

### 2.2. Design

The register and the studies based on the register information have an interventional design: patients with DM will complete questionnaires before using the FSL-FM, after six months of use, and after twelve months of use. The total follow-up period will be 1 year.

### 2.3. Population

The intended study population consists of adults (≥18 years) with both type 1 and type 2 DM who use insulin. All patients will be required to be treated by a hospital-based secondary care diabetes team, have health insurance with Zilveren Kruis/Achmea (the largest health insurance company in the Netherlands), and belong to one or more of the target groups (see Table 1).

### 2.4. Group Size Inclusion

No total group estimation through power calculation has been performed since all patients who fulfill the criteria described below will be included if considered fit for participation. Nonetheless, since some estimates can help support the drive to recruit enough participants to detect significant differences, a power calculation was performed for target group 3 (“unable to reach an HbA1c <65 mmol/mol despite maximal efforts”), for whom the important primary outcome measure is the change in HbA1c. Therefore, the sample size was calculated for HbA1c using Sample Power 3.0 for the calculation. Based on a paired *t*-test, two-sided testing was carried out, with a power of 90%. An estimated 10% reduction in HbA1c is considered clinically relevant and feasible in this target group. The correlation between the HbA1c at the start of the study and the HbA1c at 6 months was estimated at 0.80, resulting in a standard deviation of 8.854. The mean HbA1c at the baseline was estimated at 64 mmol/mol. A 10% reduction (6.4 mmol/mol) would result in an HbA1c of 57.4 mmol/mol after 6 months of follow-up. The lowest acceptable reduction of the HbA1c is 5% (3.2 mmol/mol).

#### 2.4.1. Hypotheses

  H0: *μ*Δ < 5% (3.2 mmol/mol)
  H1: *μ*Δ ≥ 5% (3.2 mmol/mol), most preferably 10% (6.4 mmol/mol)

Sample size was calculated using parametric and nonparametric analyses, yielding sample sizes of 83 and 92 participants, respectively.

### 2.5. Selection Procedure

The departments of internal medicine and/or diabetes centers of all Dutch hospitals are asked to include individuals based on the inclusion criteria as described in Table 1. All these centers have been approached in writing, and extensive information regarding the register has been provided. This information is also available in the register, and the procedure is available on http://www.FSLregister.nl [14]. Each participating hospital will provide the data to a single contact person appointed within the department of internal medicine. This person is then considered responsible for collecting the data in the center.

After providing the details requested for the register (see Tables 2 and 3) and obtaining the informed consent of the projected user, the patient creates his or her own profile to log in and is asked to complete three questionnaires on the site at 0, 6, and 12 months. These questionnaires are the SF-12 [15], the EQ-6D [16–18], and a newly created questionnaire from the DVN (see Addendum 1). Although the latter questionnaire is not validated, we have included it upon request from the DVN to allow an alternative approach from the user perspective. Finally, patients will answer questions regarding diabetes-related incidents in everyday life.  Table 4 shows an overview of the self-reported and practitioner-reported data.

### 2.6. Economic Analysis

After finishing the 1-year actual study, data gathered by the Diabetes Research Center at Isala will be combined with data from the insurance company ZK by a trusted third party to create an anonymized database to allow analysis of the economic aspects of FSL use, initially concentrating on the following healthcare costs:

- Overall costs
- Hospital costs (with specific attention to admissions related to diabetes mellitus) including outpatient expenditure
- Primary care costs
- Paramedical costs
- Medication costs
- Device-related costs
- Diabetes-related costs within these separate cost groups

### 2.7. Ethical Considerations

The protocol was approved by the Medical Ethical Committee of Isala. Furthermore, the Medical Ethical Committee decided for a non-WMO liability status (METC 16.0346). Patients will participate after informed consent is obtained, after which their e-mail addresses and contact details will be collected. Patients will be asked to accept the electronic informed consent both for permission to use the information for the study and for the later use of this information to create an anonymized database, combining registry data with health insurance data.

## 3. Discussion

The possibilities to assess glucose levels with a variety of methods have been increasing in the last few years, and the trend will continue in the future. Some of these methods will be based on probes and devices which either need to be inserted or implanted (either intravascular or interstitial) [10–13, 19–24], and some will be noninvasive, e.g., measuring glucose through the skin by Raman spectroscopy [25, 26], in lacrimal fluids [27], and in sweat [28]. However, it must be concluded that the farther the probe or device is from the blood circulation, the larger the chance that the accuracy of measurements will be lost.

Manufacturing a device with an accuracy comparable to BGM when measuring glucose concentrations in the interstitial fluid and translating such concentrations into the equivalent blood glucose concentrations is challenging, especially when no calibration is required by the manufacturer or even possible, like that of the FSL-FM.

However, even in cases of less accuracy, user satisfaction can be high, and use of a device like the FSL-FM may lead to improved metabolic control, characterized not only by a (further) decrease in HbA1c but also by maintaining an acceptable HbA1c level with less episodes of (severe) hypoglycemia.

Furthermore, from a user perspective, the classical indications for CGM use, like poor metabolic control and (frequent) occurrence of episodes of relevant hypoglycemia, might be too restrictive. In the Netherlands, use of medications like insulin, which is known to induce hypoglycemia on occasion, may lead to job loss (e.g., when driving a bus or a heavy lorry), which might be prevented when users show their ability to better control their blood glucose levels. Another reason to use FSL-GM includes being able to properly participate in sports and exercise activities, which leads not only to more user satisfaction but also to health gain in the long term.

The strength of the proposed approach lies in the inclusion of a large group of patients from all over the Netherlands, which may result in findings that are generalizable.

The limitations of this approach are inherent to the study design. Most of the results are reported by the participants themselves. Since in due course they might profit from positive results of this study by portraying results more favorably, this could introduce response bias. Nonetheless, by starting this register from a patient/user perspective, we have made a choice to consider patient-oriented outcomes as valid and acceptable.

An improvement in glycated hemoglobin can also mean an increase in HbA1c for a person with “hypoglycemia unawareness” and “unexpected hypoglycemia.” It might be useful to relax, rather than reduce, the HbA1c target in an attempt to diminish hypoglycemic incidents and possibly regain hypoglycemia awareness. Therefore, HbA1c changes will be reported per target group. Causes of unexpected hypoglycemia can also be hypoglycemic episodes during or after exercise or in case of erratic gastric emptying.

Results from this register will aid in decision making in terms of reimbursement not only in the Netherlands but also hopefully in other countries as well.

## 4. Conclusion

In conclusion, the register for the FSL-FM in the Netherlands is important because it not only is based on HbA1c, which is regarded as the sole or most important primary endpoint by regulatory agencies and insurance companies, but also emphasizes user-centered factors including health-related QoL, changes in self-confidence which may allow users to explore their physical limits more fully, device user-friendliness, and major societal aspects like work absenteeism and hospital admission rates. As such, we hope that the more holistic approach used in this registry design will become more common when assessing a device like the FSL-FM, allowing reimbursement decisions to be made not only based purely on medical arguments but also on other user-centric metrics.

## Figures and Tables

**Table 1:** Inclusion criteria for target groups.

(1) Patients with “hypoglycemia unawareness” and moderate to severe hypoglycemic episodes after an average of six or more measurements per day over the past year and despite intensive support from a diabetes team
(2) Patients with unexpected hypoglycemia after obtaining an average of six or more measurements per day over the past year and despite intensive support from a diabetes team
(3) Patients treated with insulin who, despite maximal efforts (frequent blood monitoring and proper lifestyle management) and intensive support from the diabetes team, do not reach acceptable glycemic control, as evidenced by a mean HbA1c >64 mmol/mol (8.0%) over the year preceding the inclusion
(4) Patients who have an occupation whereby loss of sensation in the fingers by frequent use of BGMs may lead to disability (e.g., musicians) and who are under other circumstances would be advised by the healthcare team to perform frequent blood glucose measurements to better control their diabetes
(5) Patients who have an occupation whereby even rarely occurring hypoglycemic episodes would lead to a situation endangering themselves and/or others (e.g., bus and lorry drivers, school teachers, and sports trainers).
(6) Patients who are already eligible for CGM according to Dutch regulations; children with type 1 diabetes (up to 18 years); pregnancy wish and pregnancy in diabetic individuals (type 1 and type 2); adults with type 1 diabetes with poor metabolic control (HbA1c >64 mmol/mol) for a longer period.
(7) Patients already using the FSL-FM at their own expense but fall into one of the categories described above

**Table 2:** Required baseline data and measurements.

(i) Demographic data including type of DM (type 1 DM, type 2 DM, latent autoimmune diabetes, maturity-onset diabetes of the young, and others)
(ii) Indication for participation
(iii) HbA1c levels of preceding year (last four measurements), to be reported by the health professional
(iv) Absence or presence of neuropathy, retinopathy, nephropathy, or macrovascular disease
(v) Frequency of SBGM
(vi) Scores of EQ-6D and SF-12v2 questionnaires
(vii) Self-reported number of diabetes-related hospitalizations in the previous year
(viii) Self-reported estimated/measured hypoglycemias 3 months before the device is used; specific categorization for severe (grade III) hypoglycemia (grade I (mild) symptoms with blood glucose <70 mg/dL, grade II (minor) symptoms with blood glucose <56 mg/dL, and grade III (severe) symptoms with glucose <40 mg/dL, requiring third-party assistance)
(ix) Self-reported levels of working day losses or reduced functioning (including sports performance) due to glucose variability
(x) DVN questionnaire

**Table 3:** Follow-up data and measurements after 6 and 12 months.

(i) Scores from EQ-6D and SF-12v2 questionnaires
(ii) HbA1c levels within the preceding 6 months, to be reported both by the user and the healthcare professional
(iii) Any diabetes-related hospitalizations
(iv) Changes in presence of complications
(v) Self-reported number of diabetes-related hospitalizations in the previous year
(vi) Self-reported estimated/felt/measured hypoglycemias in three months before filling out questionnaires; specific categorization for severe (grade III) hypoglycemia
(vii) Self-reported levels of working day losses or reduced functioning (including sports performance) due to dysregulation of DM
(viii) DVN questionnaire
(ix) Satisfaction from the use of FLS-FM

**Table 4:** Overview of self-report and practitioner-reported data.

Self-report	Reported by practitioner
(i) HbA1c T6 and T12 months	(i) Target group
(ii) QoL using the 12-Item Short-Form Health Survey (SF-12)	(ii) HbA1c T0, T6, and T12 months
(iii) Six-dimensional EuroQol instrument (EQ-6D)	
(iv) Questionnaire DVN	
(v) The number of hypoglycemic episodes	
(vi) The number of hospitalizations related to DM	
(vii) Changes in the number of working days lost/days absent from work due to illness	
(viii) Daily functioning (including sports performance)	

## Data Availability

Since this manuscript is about architecture and design, no data are available.

## Supplementary Materials

Supplementary Materials Addendum 1. Newly created questionnaire from the DVN.

## References

[1] Nathan D. M., Cleary P. A., Backlund J. Y. (2005). Intensive diabetes treatment and cardiovascular disease in patients with type 1 diabetes. *New England Journal of Medicine*.

[2] Kilpatrick E. S., Rigby A. S., Goode K., Atkin S. L. (2007). Relating mean blood glucose and glucose variability to the risk of multiple episodes of hypoglycaemia in type 1 diabetes. *Diabetologia*.

[3] Siegelaar S. E., Holleman F., Hoekstra J. B. L., DeVries J. H. (2010). Glucose variability; does it matter?. *Endocrine Reviews*.

[4] Klonoff D. C., Ahn D., Drincic A. (2017). Continuous glucose monitoring: a review of the technology and clinical use. *Diabetes Research and Clinical Practice*.

[5] Benkhadra K., Alahdab F., Tamhane S. (2017). Real-time continuous glucose monitoring in type 1 diabetes: a systematic review and individual patient data meta-analysis. *Clinical Endocrinology*.

[6] Pickup J. C., Ford Holloway M., Samsi K. (2015). Real-time continuous glucose monitoring in type 1 diabetes: a qualitative framework analysis of patient narratives. *Diabetes Care*.

[7] Schmelzeisen-Redeker G., Staib A., Strasser M., Müller U., Schoemaker M. (2013). Overview of a novel sensor for continuous glucose monitoring. *Journal of Diabetes Science and Technology*.

[8] Abbot (May 2018). FreeStyle libre. http://www.Freestylelibre.com.

[9] Fokkert M. J., Van Dijk P. R., Edens M. A. (2017). Performance of the FreeStyle Libre Flash glucose monitoring system in patients with type 1 and 2 diabetes mellitus. *BMJ Open Diabetes Research & Care*.

[10] Dexcom (February 2018). Dexcom continuous glucose monitoring. http://www.dexcom.com.

[11] Medtronic, February 2018, http://www.medtronic.com

[12] Senseonics, February 2018, http://www.senseonics.com

[13] GlySens®, February 2018, http://www.glysens.com

[14] Isala (May 2018). FreeStyle libre register. http://www.fslregister.nl.

[15] Hagell P., Westergren A., Årestedt K. (2017). Beware of the origin of numbers: standard scoring of the SF-12 and SF-36 summary measures distorts measurement and score interpretations. *Research in Nursing & Health*.

[16] Hoeymans N., van Lindert H., Westert G. P. (2005). The health status of the Dutch population as assessed by the EQ-6D. *Quality of Life Research*.

[17] Van Reenen M., Janssen B. (2015). *EQ-5D-3L User Guide: Basic Information on How to Use the EQ-5D-3L Instrument*.

[18] Lammers L. M., M Stalmeijer P. F., McDonnell J. (2005). Kwaliteit van leven meten in economische evaluaties: het Nederlands EQ-5D-tarief. *Nederlands Tijdschrift Voor Geneeskunde*.

[19] GlucoWise™, February 2018, http://www.gluco-wise.com

[20] Genexo (February 2018). Glucosense® Pro. http://genexo.eu/en/products/16-products/25-glucosense-pro.

[21] Nemaura Medical (February 2018). SugarBEAT®. http://www.nemauramedical.com/sugarbeat.

[22] Lee H., Choi T. K., Lee Y. B. (2016). A graphene-based electrochemical device with thermoresponsive microneedles for diabetes monitoring and therapy. *Nature Nanotechnology*.

[23] Bariya M., Nyein H. Y. Y., Javey A. (2018). Wearable sweat sensors. *Nature Electronics*.

[24] NovioSense, February 2018, http://www.noviosense.com

[25] Scholtes-Timmerman M. J., Bijlsma S., Fokkert M. J., Slingerland R., van Veen S. J. F. (2014). Raman spectroscopy as a promising tool for noninvasive point-of-care glucose monitoring. *Journal of Diabetes Science and Technology*.

[26] Shih W., Bechtel K. L., Rebec M. V. (2015). Noninvasive glucose sensing by transcutaneous Raman spectroscopy. *Journal of Biomedical Optics*.

[27] Kim J., Minji K., Lee M.-S. (2017). Wearable smart sensor systems integrated on soft contact lenses for wireless ocular diagnostics. *Nature Communications*.

[28] Sim J. Y., Ahn C.-G., Jeong E.-J., Kim B. K. (2018). *In vivo* microscopic photoacoustic spectroscopy for non-invasive glucose monitoring invulnerable to skin secretion products. *Scientific Reports*.

