# Supplementary material for: Use of FreeStyle Libre Flash Monitor Register in the Netherlands (FLARE-NL1): Patient Experiences, Satisfaction, and Cost Analysis
Source: Int J Endocrinol. 2019 Nov 3;2019:4649303. doi: 10.1155/2019/4649303 (PMC6875221; doi:10.1155/2019/4649303)
Supplement: Supplementary Materials — Addendum 1. Newly created questionnaire from the DVN. [file 4649303.f1.docx]

Top of form

***Addendum 1.***

***Prior to the FSL-FGM period***

Questions about blood glucose measurement with strips and meter

**1** To me, the presence of strangers when I want to measure blood glucose is:

Single answer

□ A big obstacle

□ An obstacle

□ A small obstacle

□ No obstacle

□ I’ve been using the FreeStyle Libre for a long time and can’t remember

**2** For me, deciding what to do best, based on the blood glucose level measured, is:

Single answer

□ Very difficult

□ A significant problem

□ Reasonably well possible

□ Going well

□ I’ve been using the FreeStyle Libre for a long time and can’t remember

**3** Measuring my blood glucose in a poorly illuminated space is:

Single answer

□ I don’t measure with poor illumination

□ Rather hard to do

□ Sometimes possible

□ Usually/Always

□ I’ve been using the FreeStyle Libre for a long time and can’t remember

**4** Measuring blood glucose when my hands are cold is:

Single answer

□ Seriously problematic

□ Problematic

□ Somewhat problematic

□ No problem

□ I’ve been using the FreeStyle Libre for a long time and can’t remember

**5** Measuring blood glucose if I can’t wash my hands beforehand is:

Single answer

□ A problem, so I don’t measure

□ A problem, but I still measure

□ No problem, my hands are not dirty

□ No problem, I always have something with me to clean my fingers

□ I’ve been using the FreeStyle Libre for a long time and can’t remember

**6A** Do you (usually) measure your blood glucose in a frequency you (or your practitioner) consider(s) adequate?:

Single answer

□ No, on average I measure less frequently

□ No, on average I measure more frequently

□ Yes

□ I’ve been using the FreeStyle Libre for a long time and can’t remember

**6B** If you measure less frequently than you or your practitioner think(s) you need, what is the reason?

Multiple answers are possible

□ I don’t like the painful finger prick

□ I find it is difficult to fit in with my work/activities

□ I do not want my diabetes to be visible

□ I do not have the opportunity to wash my hands

□ I do not carry my glucose testing material with me

□ I find it difficult to be confronted with the results

□ I find it difficult that high or low glucose results is stored in the memory of the meter.

□ I think it takes too much time

□ Other, .........

□ I’ve been using the FreeStyle Libre for a long time and can’t remember

**7** How often do you measure your level before participating in traffic as a driver? (Between 60 and 0 minutes before departure)

Single answer

□ Never

□ Sometimes

□ Usually

□ Always

□ n/a

□ I’ve been using the FreeStyle Libre for a long time and can’t remember

**8** How satisfied are you with the number of times you measure your glucose level?

Please give a grade from 0 to 10

□ ……………..

□ I’ve been using the FreeStyle Libre for a long time and can’t remember

**9** How often do you on average use the same lancet?

□……………..

**9B** If more than once, what is the reason?

Multiple answers possible

□ I (often) forget to take extra needles with me

□ I do not take the time to change the needle

□ Changing the needle in public attracts attention which makes me uncomfortable

□ I do not think it is necessary, as long as it performs well.

□ Other, ..............

**10** How often per month do you waste a test strip without obtaining an (accurate) glucose level.

□ ....

**10B** What is the most common cause?

Multiple answers possible

□ Glucose strip inserted incorrectly in the meter

□ Too little blood on the strip

□ Test strip does not work

□ The surroundings are too cold or too hot

□ Test strip is contaminated (e.g. it fell on the floor)

□ Meter indicates that no testing is possible

□ Test strip is over date

□ Too quick with applying blood to the test strip (before the meter was ready).

□ Other, ..................

**11** How accurate do you think the values of your glucose meter are?

Please give a score from 0 to 100

***After the FSL period (T=6, T=12)***
**1** To me, the presence of strangers while measuring my glucose levels is

*Single answer*
□ A big obstacle
□ An obstacle
□ A small obstacle
□ No obstacle

**2** When you measure in public, how obvious do you feel it is that you have (or could have) diabetes?

*Single answer*
□ Not at all
□ Somewhat
□ Very

**3** For me, deciding what to do best, based on the trend of the blood glucose levels over the previous eight hours, is:

Single answer

□ Very difficult

□ A significant problem

□ Reasonably well possible

□ Going well

□ I’ve been using the FreeStyle Libre for a long time and can’t remember

**4** Scanning my blood glucose in a poorly illuminated space is:

Single answer

□ I don’t measure with poor illumination

□ Rather hard to do

□ Sometimes possible

□ Usually/Always

□ I’ve been using the FreeStyle Libre for a long time and can’t remember

**5** How often do you scan your level before participating in traffic as a driver (between 60 and 0 minutes before departure)
*Single answer*
□ Never
□ Sometimes

□ Usually
□ Always
□ n/a

**6** By using the FreeStyle Libre, I feel
*More answers are possible*
□ More anxious
□ Insecure
□ More patient
□ Less anxious
□ Safer
□ Less patient

**7** The FSL
A Allows me to exercise

□ more / □ less

□ the same
B Allows me to understand my glucose level fluctuations

□ better /

□ worse/

□ the same
C Causes

□ more / □ less hypoglycaemia.

□ It does not make any difference
D Causes my hypoglycaemic periods to be

□ more severe /

□ less severe /

□ the same
E Causes

□ more / □ less hyperglycaemia.

□ It does not make any difference
F Is regulating my glucose around a meal

□ with greater ease /

□ with greater difficultly.

□ It does not make any difference

G Causes me to change my insulin dose

□ more often /

□ less often.

□ It does not make any difference


**8** Does the sensor irritate your skin?
**8b** Did you have to remove sensors due to irritation of your skin?
**8c** How many sensors did you have to remove earlier than expected?

**9** Did you have to remove sensors due to a technical defect?
**9b** How many sensors did you have to remove due to a technical defect?

**10** Did you have to remove sensors due to an allergy?

**10b** How many sensors did you have to remove due to an allergy?

**11** By using the Free Style Libre, my housemates are

Check the applicable answers.
□ Very worried about my glucose
□ More worried about my glucose
□ Equally worried about my glucose
□ Less worried about my glucose
□ My housemates do not interfere with my diabetes.
□ I do not have housemates

**12** The glucose levels shown by the FSL are subject to a slight delay (10 to 20 minutes).
For me, this means
□ That I also want to measure my actual blood glucose level with a strip
□ Nothing, because, it is sufficient information for me
□ Not much, because the trend displayed compensates for this

**13** A new FSL sensor gives less accurate results on the first day of use.

What would you do if you keep using the FSL?

□ I would measure extra with a glucose meter on the first day
□ If the FSL is too low or too high, I measure once more with a blood glucose meter.
□ I leave it for what it is and just guess that day
□ This was not the case for me, my FSL was accurate right from the first day


**14** How accurate do you think the levels measured by your FSL are?
Please give a score from 0 to 100

**15** What do you think of wearing the FSL sensor continuously on your arm?
□ Very annoying
□ Annoying
□ A little bit annoying
□ No problem at all

**16** How likely are you to recommend the FSL to other insulin users?
□ Very likely
□ Likely
□ Somewhat likely
□ Unlikely
□ Very unlikely

The following questions are for participants who are used to always wear their glucose sensor.
**17**
The FSL has no alarm sounds for levels that are too high or too low, or rapid glucose drops or increases
Is this an objection for you to switch to the FSL?
□ Yes
□ No

**17B**
Could this be a reason for you to switch to the FSL?
□ Yes
□ No

Please list your experiences with the FSL below

…………………………….

Did you miss any questions?

………………………

Do you have any information that you think is important with regard to this study?

…………………….
